# The VLPFC-Engaged Voluntary Emotion Regulation: Combined TMS-fMRI Evidence for the Neural Circuit of Cognitive Reappraisal

**DOI:** 10.1523/JNEUROSCI.1337-22.2023

**Published:** 2023-08-23

**Authors:** Zhenhong He, Sijin Li, Licheng Mo, Zixin Zheng, Yiwei Li, Hong Li, Dandan Zhang

**Affiliations:** ^1^Institute of Brain and Psychological Sciences, Sichuan Normal University, Chengdu, 610066, China; ^2^School of Psychology, Shenzhen University, Shenzhen, 518060, China; ^3^Shenzhen-Hong Kong Institute of Brain Science, Shenzhen, 518055, China

**Keywords:** emotion regulation, reappraisal, transcranial magnetic stimulation, ventrolateral prefrontal cortex, ventromedial prefrontal cortex

## Abstract

A clear understanding of the neural circuit underlying emotion regulation (ER) is important for both basic and translational research. However, a lack of evidence based on combined neuroimaging and neuromodulation techniques calls into question (1) whether the change of prefrontal-subcortical activity intrinsically and causally contributes to the ER effect; and (2) whether the prefrontal control system directly modulates the subcortical affective system. Accordingly, we combined fMRI recordings with transcranial magnetic stimulation (TMS) to map the causal connections between the PFC and subcortical affective structures (amygdala and insula). A total of 117 human adult participants (57 males and 60 females) were included in the study. The results revealed that TMS-induced ventrolateral PFC (VLPFC) facilitation led to enhanced activity in the VLPFC and ventromedial PFC (VMPFC) as well as attenuated activity in the amygdala and insula during reappraisal but not during nonreappraisal (i.e., baseline). Moreover, the activated VLPFC intensified the prefrontal-subcortical couplings via the VMPFC during reappraisal only. This study provides combined TMS-fMRI evidence that downregulating negative emotion involves the prefrontal control system suppressing the subcortical affective system, with the VMPFC serving as a crucial hub within the VLPFC-subcortical network, suggesting an indirect pathway model of the ER circuit. Our findings outline potential protocols for improving ER ability by intensifying the VLPFC-VMPFC coupling in patients with mood and anxiety disorders.

**SIGNIFICANCE STATEMENT** Using fMRI to examine the TMS effect, we uncovered that the opposite neural changes in prefrontal (enhanced) and subcortical (attenuated) regions are not a byproduct of emotion regulation (ER); instead, this prefrontal-subcortical activity per se causally contributes to the ER effect. Furthermore, using TMS to amplify the neural changes within the ER circuit, the “bridge” role of the VMPFC is highlighted under the reappraisal versus nonreappraisal contrast. This “perturb-and-measure” approach overcomes the correlational nature of fMRI data, helping us to identify brain regions that causally support reappraisal (the VLPFC and VMPFC) and those that are modulated by reappraisal (the amygdala and insula). The uncovered ER circuit is important for understanding the neural systems underlying reappraisal and valuable for translational research.

## Introduction

Emotion regulation (ER) is essential for mental health and adaptive social behaviors (Eftekhari et al., 2009). ER entails neurobiological processes from cortical control systems (i.e., prefrontal regions) that modulate the activity of subcortical affective response systems (e.g., amygdala and insula) (Frank et al., 2014; Zilverstand et al., 2017). Gross and colleagues (Gyurak et al., 2011; Braunstein et al., 2017) proposed that ER could be automatic/implicit or voluntary/explicit. While automatic ER is evoked unconsciously by affective stimuli and mainly engages the medial prefrontal cortex (MPFC) (Etkin et al., 2015), voluntary ER requires conscious effort and is launched and maintained by the lateral PFC (LPFC) (Frank et al., 2014; Morawetz et al., 2017).

Among various voluntary ER strategies, cognitive reappraisal can effectively reduce negative experiences and emotion-related neural responses with long-lasting benefits (Buhle et al., 2014; McRae and Gross, 2020). Cognitive reappraisal is mainly supported by the dorsolateral PFC (DLPFC), ventrolateral PFC (VLPFC), and ventromedial PFC (VMPFC) (Kohn et al., 2014; Morawetz et al., 2017). The DLPFC and VLPFC are essential in launching ER but may have different functions: the former maintains multiple appraisals for the current affective situation in working memory, while the latter is responsible for selecting the appropriate appraisal and inhibiting others in accordance with the ER goal (Silvers and Moreira, 2019). Transcranial direct current or magnetic stimulation (tDCS or TMS) studies show that the DLPFC and VLPFC play a causal role in implementing reappraisal (Feeser et al., 2014; Marques et al., 2018). However, most of our knowledge on the mechanisms underlying ER stems from neuroimaging studies that are inherently correlational, supplemented by limited causal clues from neuromodulation studies (mainly highlighting the causal role of the LPFC at the behavioral level). Previous research has not examined the influence of the LPFC on downstream neural circuitry specifically during reappraisal. Therefore, the current study aims to use a combined neuromodulation and observation approach to investigate whether the downregulation of negative emotion is achieved through the LPFC's suppression of the subcortical affective system.

Additionally, the pathways linking the prefrontal top-down control system and subcortical affect system remain unclear. Structural imaging evidence suggests the existence of interconnected pathways between the LPFC, VMPFC, and subcortical regions underlying ER (M. J. Kim et al., 2011; Wilcox et al., 2016), but the specific functional reactions of the VMPFC within this neural circuit are still a subject of debate. The direct-pathway model assumes that prefrontal regions directly modulate subcortical regions during ER implementation (Ochsner et al., 2012). Indeed, negative emotion reduction during reappraisal was associated with direct input from the VLPFC to the amygdala and ventral striatum (Wager et al., 2008) and a direct pathway from the DLPFC to the ventral striatum was identified in cognitive regulation of substance craving (Kober et al., 2010). Moreover, a meta-analysis conducted by Buhle et al. (2014) demonstrated that the VMPFC activation during reappraisal is not consistent across studies, suggesting that the VMPFC may not be essential for reappraisal. Alternatively, the indirect-pathway model proposes that voluntary ER is supported by prefrontal modulation of the subcortical responses via their mutual connections to the VMPFC (Schiller and Delgado, 2010; Diekhof et al., 2011; Etkin et al., 2011). Indeed, the VMPFC mediated the connections between the VLPFC and amygdala during reappraisal (Johnstone et al., 2007), and there is evidence that reappraising fearful situations engaged LPFC regions, which modulated amygdala activity via the VMPFC (Urry et al., 2006; Delgado et al., 2008). The mediating role of the VMPFC in the LPFC input to the amygdala has also been highlighted in dynamic causal modeling (DCM) studies (Steward et al., 2021). However, because of the correlational nature of neuroimaging studies, it remains inconclusive whether the top-down prefrontal modulation projects directly to the subcortical regions or indirectly via the VMPFC. TMS can be used to gain insight into the downstream effects of neural activity following superficial prefrontal stimulation. It is supposed that high-frequency TMS is generally known to produce an excitatory effect on neural activity, while low-frequency TMS is known to generate an inhibitory effect (Klomjai et al., 2015).

Thus, we combined TMS and fMRI to shed light into the neural pathway from the prefrontal control system to the subcortical affective system. TMS was used to temporally enhance the excitability of the LPFC and examine causality in the neural circuit of reappraisal. We evaluated ER in negative social scenarios because painful social emotions are an important part of the human experience and are constantly faced and regulated in our everyday lives (He et al., 2020a, b; Nasso et al., 2022). The right VLPFC was selected as the target of neuromodulation because it has been consistently activated in voluntary ER regardless of strategies (e.g., reappraisal, distraction) and regulatory goals (i.e., upregulation or downregulation) (Kohn et al., 2014; Morawetz et al., 2017) and because of its essential role in automatic (Riva et al., 2012) and voluntary (Zhao et al., 2021; S. Li et al., 2022) downregulation of social pain.

Two hypotheses were formulated regarding the neural underpinnings of voluntary ER. First, assuming that the VLPFC is an essential region in voluntary emotion regulation, TMS facilitation would launch a chain reaction in the neural circuit of reappraisal: increased activity in the TMS target (i.e., VLPFC) and attenuated activity in subcortical regions of the amygdala and insula, which are responsible for generating social pain (Rotge et al., 2015; Mwilambwe-Tshilobo and Spreng, 2021). Second, we predicted that the prefrontal-subcortical connectivity should increase as a result of VLPFC facilitation. However, we cannot predict *a priori* whether the VMPFC plays a hub role in the prefrontal-subcortical pathway because of the available conflicting evidence. Thus, we empirically tested whether VMPFC activity and connectivity were influenced by VLPFC TMS. Accordingly, the ROIs were the right VLPFC, bilateral VMPFC, amygdala, and insula. The bilateral amygdala and insula were chosen as they are representative hubs in the subcortical affective system (Berntson et al., 2011) and have been particularly associated with social pain processing (Zovetti et al., 2021).

## Materials and Methods

### Participants

This study included two TMS groups: the active and the sham stimulation (see Repetitive TMS (rTMS)). During the experiment design, we conducted *a priori* power analysis using G*Power 3.1.9 (*F* tests, ANOVA: repeated-measures, within-between interaction) based on the effect size ($\eta_{p}^{2}$ = 0.083) reported in our recent TMS study (Zhao et al., 2021). According to this power analysis, 26 participants would ensure 80% statistical power. However, 13 participants per group is considered a small sample size in present-day neuroscience studies focusing on a nonpatient population. Thus, 60 participants for each TMS group was the goal, ensuring a statistical power near 100%. In total, 120 healthy college students were recruited from Shenzhen University, and were then randomly assigned into the TMS groups. All participants were right-handed with normal or corrected-to-normal vision.

Two participants were excluded because of excessive head movements during MRI scanning, and another one failed to complete the experiment because of TMS discomfort. As a result, 58 and 59 students were included in the sham and active TMS groups, respectively. Participants in the two groups did not significantly differ in gender (male/female), age, levels of depression, anxiety, habitual use of reappraisal strategy, rejection sensitivity, and empathy ability (Beck et al., 1996), anxiety (Spielberger et al., 1983; Liebowitz, 1987), habitual use of reappraisal strategy (Gross and John, 2003), rejection sensitivity (Downey and Feldman, 1996), and empathy ability (Davis, 1980; Table 1). The study protocol was approved by the Ethics Committee of Shenzhen University. Participants signed an informed consent form before the experiment began.

**Table 1.** Demographic characteristics of the two groups

Item	Sham (*n* = 58)	Active (*n* = 59)	*t^a^*	*p*
Handedness (right/left)	58/0	59/0		
Gender (male/female)	28/31	29/29	χ^2^ = 0.08	0.783
Task order (no-reappraisal first/reappraisal first)	29/29	30/29	χ^2^ = 0.01	0.919
Age (yr)	20.21 (0.22)	20.54 (0.24)	−1.03	0.303
BDI-II	6.48 (0.80)	8.08 (0.90)	−1.33	0.187
STAI-T	39.67 (1.10)	41.56 (0.93)	−1.32	0.191
LSAS	38.02 (2.69)	43.22 (2.62)	−1.39	0.168
ERQ-R	31.41 (0.64)	30.92 (0.50)	0.62	0.537
RSQ	10.31 (0.38)	10.08 (0.35)	0.44	0.659
IRI	51.36 (1.43)	53.27 (1.37)	−0.96	0.338

Data are mean ± SE. Participants completed six questionnaires before the experimental tasks, including the following: Beck Depression Inventory, Ed 2 (BDI-II) (Beck et al., 1996); the Trait form of Spielberger's State-Trait Anxiety Inventory (STAI-T) (Spielberger et al., 1983); the Liebowitz Social Anxiety Scale (LSAS) (Liebowitz, 1987); the Emotion Regulation Questionnaire Reappraisal (ERQ-R) (Gross and John, 2003); the Rejection Sensitivity Questionnaire (RSQ) (Downey and Feldman, 1996); and the Interpersonal Reactivity Index (IRI) (Davis, 1980).

*^a^*Independent-samples *t* test (two-tailed).

### Experimental design

**Figure 1.**
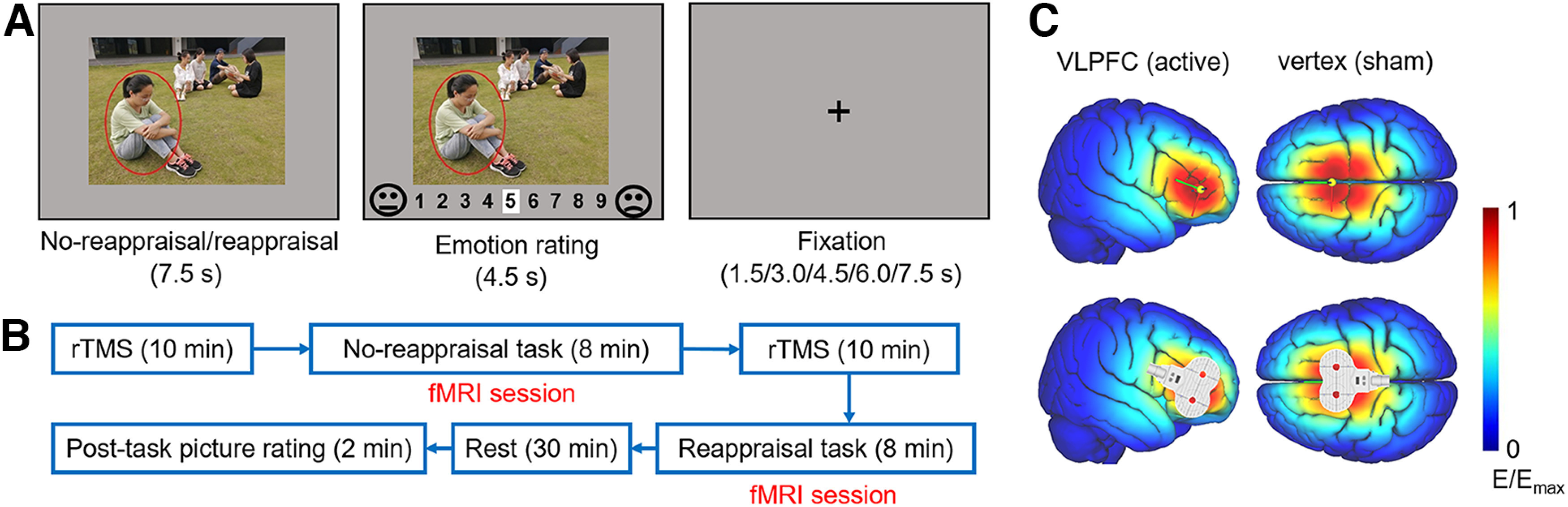
Experimental design. ***A***, Stimulus presentation in an experimental trial. For the sake of copyright, the persons in the sample image are graduate students from the research group who consented for this material to appear in academic journals. ***B***, Experimental procedure. The order of the no-reappraisal and reappraisal tasks was counterbalanced across participants. This figure shows the task order in half of the participants, that is, the no-reappraisal session ran first, followed by the reappraisal session. ***C***, The TMS electric fields in the two TMS groups (SimNIBS software, www.simnibs.org). The right VLPFC (*x* = 38, *y* = 34, *z* = −14; for active TMS group) or vertex (*x* = 0, *y* = 0, *z* = 80; for sham TMS group) was targeted. Colors represent the electric field strength scaled from 0 (blue) to the individual maximums (red).

Sixty social exclusion pictures were selected from the Social Inclusion and Exclusion in Asian Young Adults image database (Zheng et al., 2022), developed by our laboratory and successfully used to evoke social pain in previous studies (He et al., 2018, 2020a, 2020b; Zhao et al., 2021; S. Li et al., 2022). Each picture depicts a scenario of social exclusion, consisting of a rejected individual with sad or upset facial and body expressions and three or four rejecters who are talking and/or laughing together (Fig. 1*A*).

The experiment followed a 2 (regulation type: no-reappraisal vs reappraisal) × 2 (TMS group: sham vs active) mixed design. Regulation type was the within-subject factor, and TMS group was the between-subject factor. The task was divided into two sessions, corresponding to the two regulation types. The order of the no-reappraisal session and reappraisal session was counterbalanced across participants (59 participant underwent the no-reappraisal session first, and 58 participants underwent the reappraisal session first). There were no significant differences observed in the number of participants who completed each counterbalancing order within each TMS group (Table 1). Furthermore, the order factor did not show any significant influence on the dependent variables in this study. The 60 images were randomly assigned into the two sessions, with each session containing 30 images.

Each session began with a 3 s fixation cross. As shown in Figure 1*A*, a trial began with an image presentation for 7.5 s, during which participants were required to either watch passively (i.e., the no-reappraisal session) or downregulate their negative emotions using reappraisal strategies (i.e., the reappraisal session). Participants were then asked to report their negative feelings on a 9 point scale (1, not negative at all; 9, the maximum negativity). In the emotion rating interface, the white cursor first appeared in the middle of the rating scale (i.e., at 5). Participants could use their right thumb to move the cursor left or right within 4.5 s via two buttons on the response box. Jitter duration between two trials was randomly set at 1.5, 3.0, 4.5, 6.0, or 7.5 s.

At the beginning of the no-reappraisal session, participants were instructed as follows: “In this section, please think about how you would feel in the situation of the highlighted person in the picture.” At the beginning of the reappraisal session, participants were instructed as follows: “In this section, please explain the negative situation you are experiencing in a less negative way (Ochsner et al., 2002). For example, you could imagine that the group of people who are interacting with one another are talking about something that you (i.e., the highlighted person) are not interested in or you are able to make some changes and join the group very soon. Report your feelings after you reinterpret the nature of this scene.”

The experimental procedure is shown in Figure 1*B*. Participants underwent two 10 min TMS sessions, each followed by one task session (fMRI session). The duration of each TMS session was set to 10 min to ensure that the after-effects of TMS would fully cover the subsequent fMRI task session. The reason for selecting a 10 min TMS session is supported by evidence suggesting that such a session is expected to produce after-effects lasting at least 10 min (Thut and Pascual-Leone, 2010; Klomjai et al., 2015). We used offline, instead of online, TMS to avoid potential technical problems of concurrent TMS-fMRI (Bergmann et al., 2021) and reduce any side effects that may impact participants' task performances (e.g., muscle twitching). After the experimental tasks, participants were released from the scanner and allowed to relax for 30 min. They then rated the valence and arousal of 10 social exclusion pictures (5 were randomly selected from the no-reappraisal session and the other 5 from the reappraisal session) on a 9 point scale (valence: 1, the maximum negativity; 5, neutral; 9, the maximum positivity; arousal: 1, low arousal; 9, high arousal). This postscanning task explored the prolonged effect of TMS-facilitated ER half an hour after the reappraisal took place (He et al., 2020b; Zhao et al., 2021).

### Repetitive TMS (rTMS)

This study used a figure-eight-shaped coil that was connected to the magnetic stimulator (M-100 Ultimate, Shenzhen Yingchi Technology). The coil was placed in a tangential position directly over the target sites on the scalp for each participant (Berman et al., 2000; Mutanen et al., 2013). TMS was applied over the right VLPFC in the active TMS group and over the vertex to provide a similar scalp sensation in the sham TMS group. To locate the right VLPFC, we adopted the MNI coordinates (*x* = 38, *y* = 34, *z* = −14) as the activation peak in the context of social pain as identified in a recent meta-analysis (Mwilambwe-Tshilobo and Spreng, 2021). This meta-analysis investigated brain activity in response to social pain and found that the right VLPFC reliably engaged during social pain processing. To locate the vertex, the coordinate (*x* = 0, *y* = 0, *z* = 80) was determined as the midpoint of a region halfway between the nasion and inion, and equidistant from the left and right ear (W. Li et al., 2021). To locate the motor area used for the measurement of the resting motor threshold (rMT), we determined the coordinate of the left motor cortex (*x* = −38.3, *y* = −15.2, *z* = 67.9), which was found to be the optimal coil position for motor cortex stimulation (Bungert et al., 2017). Before stimulation, a T1-weighted structural MRI was obtained from each participant and normalized to the MNI space. The voxels corresponding to the location of the right VLPFC, the vertex, and the left motor cortex were marked on the participants' normalized T1 images. The location of the TMS coil was guided by a frameless stereotactic neuronavigation system (Brainsight, Rogue Research). During stimulation, the position and orientation of the coil were monitored continuously by the neuronavigational system.

Each participant's rMT was measured from their motor cortex. Three electrodes were fixed on their right palm to collect motor evoked potentials. The rMT was defined as the lowest intensity evoking at least five motor evoked potential responses with amplitudes >50 μV in 10 trials. We were able to find the hot spot (i.e., the finger motor cortex) in 116 (97%) of the 120 participants within 10 min (Julkunen et al., 2009). For these participants, the rTMS was applied at 90% of each participant's rMT during the experiment. For the other 4 participants (2 were assigned to the sham group, and the other 2 were assigned to the active group) whose finger motor cortex could not be localized during the initial attempt, we asked them to visit the laboratory for a second time and their stimulus magnetic pulses were delivered at the lower 95% CI of the average rMT of the other 116 participants (Rossi et al., 2009). The rTMS was administered at 10 Hz; this frequency could produce an excitatory effect on the target brain region according to the majority of TMS literature (Rossi and Rossini, 2004; Dayan et al., 2013). Each 10 min session contained 20 trains, each lasting 3.9 s with an intertrain interval of 26.1 s (Zhao et al., 2021). The TMS simulated electric field is illustrated on an adult brain model in Figure 1*C* (SimNIBS software, www.simnibs.org). To reduce the potential effect of body movement (especially walking) on TMS-induced neural plasticity, participants sat beside the scanner bed when receiving TMS pluses. They moved slowly from their chair to the MRI scanner bed immediately after each TMS session, and moved slowly from the scanner bed to the chair to receive TMS stimuli after the first MRI session. The time elapsed between the TMS and MRI sessions was 57.8 ± 11.6 s.

### Image acquisition

Brain images were collected using a 3-T MR scanner (Siemens Trio). Functional images were collected using an EPI sequence (number of slices, 72; gap, 0.6 mm; slice thickness, 2.0 mm; TR, 1500 ms; TE, 30 ms; flip angle, 75°; voxel size, 2 mm × 2 mm × 2 mm; FOV, 192 mm × 192 mm). Structural images were acquired through 3D sagittal T1-weighted MPRAGE (224 slices; TR, 1900 ms; TE, 2.23 ms; voxel size, 1.1 mm × 1.1 mm × 1.1 mm; flip angle, 8°; inversion time, 904 ms; FOV, 220 mm × 220 mm).

#### Image processing and statistical analysis

##### Brain activity analysis

Images were preprocessed and analyzed using Statistical Parametric Mapping (SPM12; http://www.fil.ion.ucl.ac.uk/spm). The first 10 volumes were discarded because of signal equilibration and participants' adaptation to scanning noise. All remaining images were slice time-corrected and realigned for motion correction by registration to the mean image. Artifact detection was conducted using the Artifact Detection Tools software (nitrc.org/projects/artifact_detect); global mean intensity (>2 SDs from mean image intensity for the entire scan) and motion (>2 mm) outliers were identified and entered as regressors of no interest in the first-level GLM (Stoodley et al., 2012). Then, functional images were coregistered with the T1-weighted 3D images, normalized to MNI space, and smoothed with an 8 mm FWHM isotropic Gaussian kernel.

Preprocessed data were analyzed as an event-related design in the context of the GLM approach. At the first level, we specified GLMs with two regressors for the two conditions (no-reappraisal and reappraisal). The no-reappraisal or reappraisal duration was defined as the image exposure period (7.5 s) of each trial, which was modeled as a single event convolved with the canonical HRF. To account for variance caused by head movement, six realignment motion parameters (three translations/rotations) and outlier scans identified by the Artifact Detection Tools toolbox were included as nuisance regressors. The regressors were convolved with the canonical HRF in SPM12. Each normalized image was then high-pass filtered using a cutoff time constant of 128 s. Contrast images of brain activity were produced for each participant, including (1) no-reappraisal and (2) reappraisal, using the implicit baseline as calculated by SPM.

The contrast images were taken to the second-level ROI analysis. We focused on four ROIs: the right VLPFC, bilateral VMPFC, amygdala, and insula. All ROIs were defined anatomically based on the automated anatomic labeling atlas 3 (Rolls et al., 2020). Anatomical ROI selection is a standard approach in the field for studying ER (e.g., S. H. Kim and Hamann, 2007; Denny et al., 2015), which allows for consistency across studies and makes it easier to compare results. A full-factorial analysis was performed on each ROI to test for main effects and interactions, with regulation type (no-reappraisal, reappraisal) and TMS group (sham, active) as factors (*p* < 0.012; Bonferroni-adjusted accounting for the four ROIs). For the ROI-level analyses, we extracted the averaged BOLD signals (parameter estimates) of all voxels within each ROI for each participant using the MarsBaR function (Matthew et al., 2002). The averaged parameter estimates were then submitted to the ANOVAs and plotted in bar graphs. In addition, a voxel-wise analysis was conducted within each ROI specifically for the brain activation maps, and the surviving voxels (after performing False Discovery Rate (FDR) correction across all the voxels within the ROI) are displayed on the maps.

Brain activity showing significant main effects of regulation type was used to identify the common activated regions in the two groups. These group-level results were used to locate the group maxima (i.e., the peak voxel) within the ROIs. The no-reappraisal > reappraisal contrast was used to identify group maxima in the amygdala and insula, while the reappraisal > no-reappraisal contrast was used to identified group maxima in the right VLPFC and VMPFC. These group maxima were then used exclusively to determine the volumes of interest (VOIs) for the DCM model space, and were not used for the analyses of overall activation.

For completeness, we also conducted a full-factorial analysis on the whole-brain level, with regulation type and TMS group as factors. For both the ROI and whole-brain analyses, participants' age and gender were included as covariates. Statistical maps were corrected for multiple comparisons using the FDR (*p* < 0.05).

##### DCM

DCM was used because it allows for testing competing models to determine the direction and nature of information flow (Friston et al., 2019). We specifically aimed to test whether the modulatory influence of reappraisal on prefrontal-subcortical pathways crucially involves the VMPFC.

DCM is a model-based effective connectivity analysis which provides a Bayesian framework for making inferences about causal relationship between interacting cortical regions (Friston et al., 2003). For a given model, DCM allows modeling of the endogenous connections among brain areas, which is context-independent (i.e., intrinsic connections). The impact of experimental stimuli can be modeled directly on specific regions (i.e., driving inputs) or on the strength of coupling between two regions (i.e., modulatory inputs). These parameters are expressed in Hz within the DCM framework.

First, we selected VOIs and extracted time-series. We considered four regions: the VLPFC, VMPFC, amygdala, and insula. Given that only commonly activated regions in the two groups can be included in DCM (Seghier et al., 2010), the contrast map of the main effect of regulation type from the full-factorial ROI analyses (no-reappraisal > reappraisal & reappraisal > no-reappraisal) was summarized (see Table 3). This group-level result was used to locate the group maxima of the predefined regions. Reappraisal > no-reappraisal contrast identified group maxima (*x* = 38, *y* = 18, *z* = 26) in the right VLPFC and the right VMPFC (*x* = 4, *y* = 40, *z* = −16), which was very close to the activated regions reported in previous studies focusing on ER (Ochsner et al., 2012; Kohn et al., 2014). No-reappraisal > reappraisal contrast identified group maxima (*x* = 30, *y* = 0, *z* = −24) in the right amygdala and the left insula (*x* = −30, *y* = 24, *z* = 6), close to the deactivated regions reported in previous ER studies (Frank et al., 2014). Subject-level maxima of each region (except the amygdala) was constrained to be a maximum of 8 mm from the group maxima, and exceeded a liberal statistical threshold of uncorrected *p* < 0.05 (Zeidman et al., 2019). Each VOI (except the amygdala) was then defined as a 6-mm-radius sphere centered at the identified subject-level maxima, including all the voxels surviving the threshold of uncorrected *p* < 0.05. For the amygdala VOI, peak voxel was constrained to be within the corresponding anatomic mask because amygdala is such a small region that a 6-mm-spherical VOI would likely include nonamygdala voxels in proximity (Koush et al., 2019). Time-series were then extracted from VOIs at the subject level, adjusted for the *F* contrast of effects of no-interests to remove head motion. Based on these time courses, DCM was used to model the effective connectivity between VOIs.

**Figure 2.**
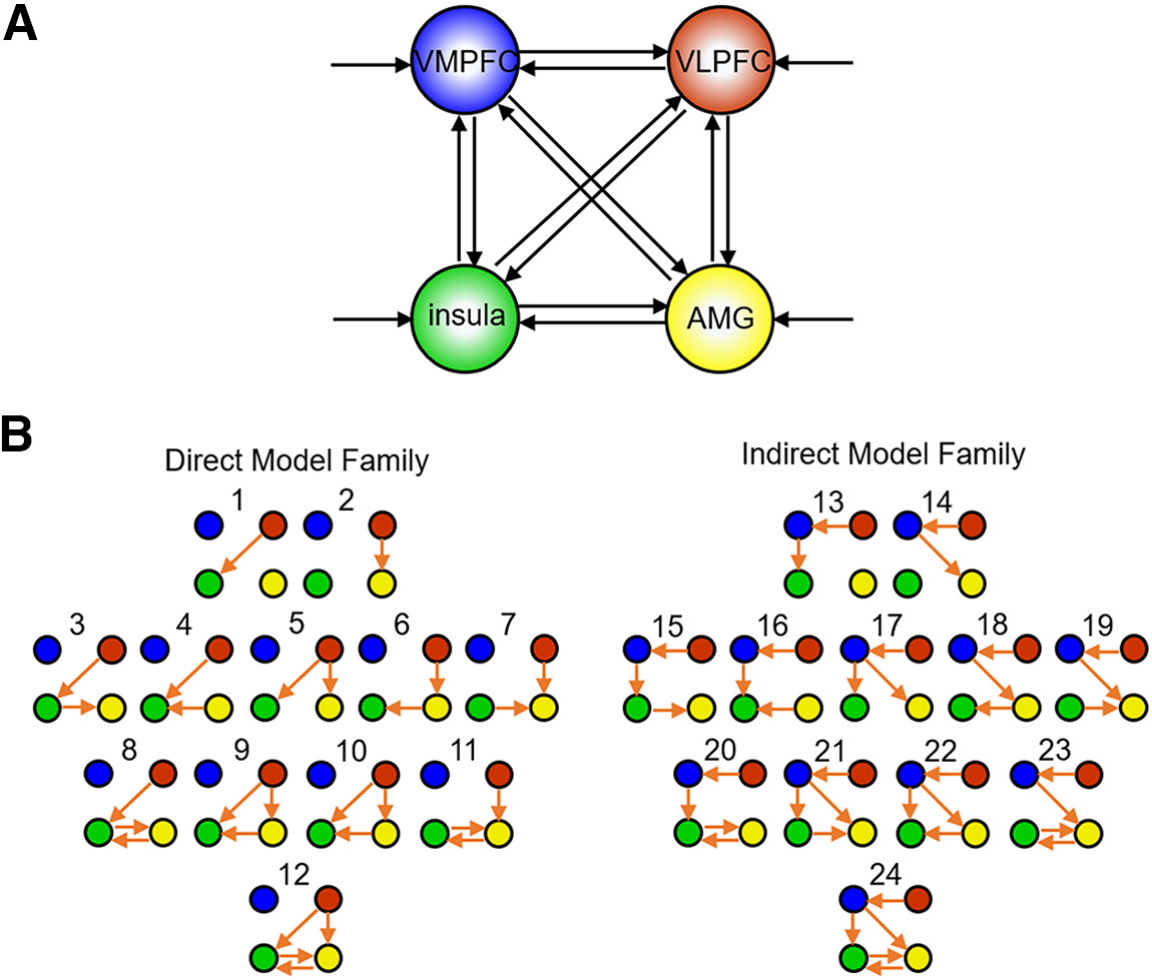
Model space. ***A***, Illustration of the estimated model. The nodes comprising the model are the right VLPFC (red), right VMPFC (blue), right amygdala (AMG; yellow), and left insula (green). Solid lines with arrows indicate the bidirectional intrinsic connections and driving inputs. ***B***, Two model families with two different core pathways modulated by the ER effect. Among the 24 different models, models 1-12 constitute the direct model family with VLPFC-to-subcortical connections as core pathways, while models 13-24 constitute the indirect model family with VLPFC-to-VMPFC-to-subcortical connections as core pathways. All models have the same intrinsic connections and driving inputs. Orange arrows indicate the ER modulated connections. Models are arranged in respect to their complexity: models in the first row (1, 2) include 1 or 2 core modulatory connections; models in the second and third row (3-11, 15-23) include 1 or 2 core modulatory connections, combined with 1 or 2 subcortical connections; models in the last row (12 and 24) represent the most complex models with all possible modulatory effects. Driving input (image presentation) enters all nodes in all models.

Second, based on VOI time courses, the model space was constructed, including two model families (direct and indirect) with different core pathways modulated by ER (Fig. 2). We assumed bidirectional intrinsic connections between all the four regions (i.e., right VLPFC, right VMPFC, right amygdala, and left insula; Fig. 2*A*). Visual events in the task (image presentation) were used as driving input, directed to all regions. The ER effect (reappraisal vs nonreappraisal) was used as modulatory input. Model space was constructed and divided into two model families with two different core pathways modulated by ER: while the modulatory input exerted its influence via the VLPFC-to-subcortical (amygdala or insula) connections in the direct model family, the ER modulatory input exerted its influence via the VLPFC-to-VMPFC-to-subcortical connections in the indirect model family. In addition to these core pathways, we also assumed that the ER effect might modulate the interconnectivity between amygdala and insula (Cohen Kadosh et al., 2016). Therefore, within each of the model families, the modulatory effect of ER could be modeled in 12 possible variants (Fig. 2*B*).

Third, the models were compared on both the family level and within a winning family to select an optimal model that fitted the data best. For each participant, all 24 models differed in respect to the modulatory effect (Fig. 2*B*, orange arrows), but they had the same endogenous connections and driving input within one family. Random-effects Bayesian model selection (implemented in SPM12) was used (Stephan et al., 2009) to compare families of models and to find the optimal model for each TMS group (active, sham). At the family level, we computed the exceedance probability of each family of models to select the optimal family from several candidate families. At the model level, we computed the exceedance probability of each model to select the optimal model from a group of candidate models within the winning family.

Fourth, Bayesian model averaging was used to calculate weighted-model parameters of the winning model (Kasess et al., 2010). Parameters (connection strengths) were then submitted to (1) one-sample *t* tests to reveal subgroup effects that had the greatest evidence of being non-zero and (2) independent-samples *t* tests to reveal between-group differences. The results were reported as posterior probabilities for each possible connectivity pathway (Zeidman et al., 2019). Significant effects at the subgroup level or between-group level are reported using an FDR-corrected *p* value for multiple comparisons.

##### Brain-behavioral correlations

For each participant, averaged BOLD signals (parameter estimates) were extracted within each ROI using the MarsBaR function (Matthew et al., 2002). We calculated two-tailed Pearson's correlations between the participant's reappraisal success (difference between subjective rating of negative feelings in the no-reappraisal and reappraisal sessions) (Wager et al., 2008) and the difference between their β values (averaged BOLD signals within each AAL ROI) for the reappraisal and no-reappraisal sessions. Correlations were calculated separately for each ROI from the different groups. Statistical significance was set at *p*(FDR) < 0.05.

### Data and code availability

The data and code of this study would be available on reasonable request and with approvals of Institute of Brain and Psychological Sciences, Sichuan Normal University. More information on making this request can be obtained from the corresponding author (D.Z.).

## Results

### Behavioral results

**Figure 3.**
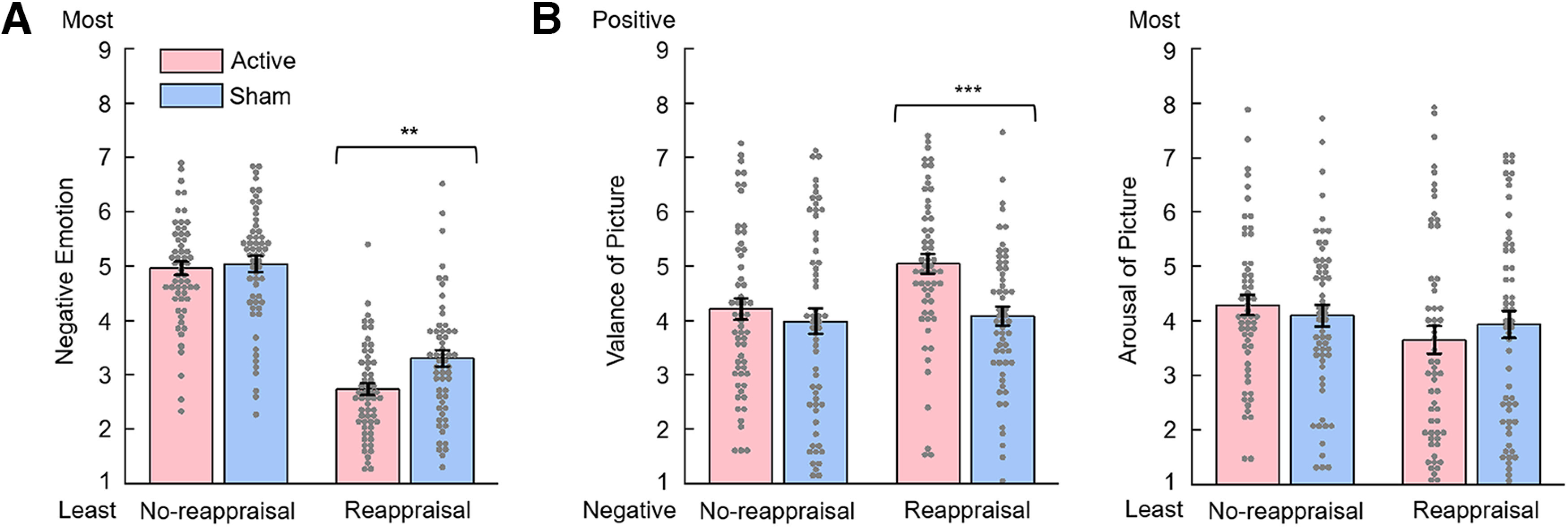
Ratings of negative emotions and post-task picture ratings in the active and sham TMS groups. ***A***, Ratings of negative emotions during the emotional regulation task (1 for the least negative and 9 for the most negative). ***B***, Postscanning ratings of valence (1 for the most negative and 9 for the most positive) and arousal of pictures (1 for the least arousing and 9 for the most arousing). Error bars indicate SEM. **p < 0.01. ***p < 0.001.

The data being reported in the text can be found in Figure 3.

#### Ratings of negative emotion

Subjective ratings of negative emotions were examined using two-way mixed-design ANOVA (regulation type × TMS group). The most important finding was the two-way interaction between TMS group × regulation type (*F*_(1,115)_ = 5.57, *p* = 0.020, $\eta_{p}^{2}$ = 0.046) (Fig. 3*A*). A simple effects analysis indicated that, while the active TMS group (2.72 ± 0.86; mean ± SD) reported lower negative feelings than the sham TMS group (3.32 ± 1.10) in the reappraisal session (*F*_(1,115)_ = 10.79, *p* = 0.001, $\eta_{p}^{2}$ = 0.086), no significant difference between the groups' ratings was observed in the no-reappraisal session (*F*_(1, 115)_ = 0.07, *p* = 0.795, $\eta_{p}^{2}$ = 0.001; 4.97 ± 0.94 and 5.02 ± 1.10 in the active and sham groups, respectively).

The main effect of regulation type was highly significant (*F*_(1,115)_ = 290.42, *p* < 0.001, $\eta_{p}^{2}$ = 0.716), with participants reporting fewer negative feelings in the reappraisal (3.02 ± 1.02) than in the no-reappraisal (5.00 ± 1.02) sessions. Additionally, there was a significant main effect of TMS group (*F*_(1,115)_ = 4.97, *p* = 0.028, $\eta_{p}^{2}$ = 0.041), with participants reporting fewer negative feelings in the active (3.85 ± 0.70) than in the sham (4.17 ± 0.86) TMS groups.

#### Postscanning picture ratings

Subjective ratings of valence of the pictures (selected from the no-reappraisal and reappraisal sessions) were examined using the two-way mixed-design ANOVA (regulation type × TMS group). The most important finding was the two-way interaction between TMS group × regulation type (*F*_(1,115)_ = 4.20, *p* = 0.043, $\eta_{p}^{2}$ = 0.035) (Fig. 3*B*). A simple effects analysis indicated that, while the active TMS group (5.04 ± 1.37; mean ± SD) reported higher valance of the pictures than the sham TMS group (4.11 ± 1.29) in the reappraisal session (*F*_(1,115)_ = 14.40, *p* < 0.001, $\eta_{p}^{2}$ = 0.111), no significant difference in valance ratings was observed between the groups in the no-reappraisal session (*F*_(1,115)_ = 0.77, *p* = 0.382, $\eta_{p}^{2}$ = 0.007; sham = 4.22 ± 1.48 and active = 3.95 ± 1.80). The main effect of regulation type was highly significant (*F*_(1,115)_ = 9.03, *p* = 0.003, $\eta_{p}^{2}$ = 0.073): participants reported higher valance of pictures in the reappraisal (4.58 ± 1.40) than those in the no-reappraisal (4.09 ± 1.65) session. Additionally, there was a significant main effect observed in TMS group (*F*_(1,115)_ = 7.15, *p* = 0.009, $\eta_{p}^{2}$ = 0.059): participants in the active group (4.63 ± 1.23) reported higher valance of pictures than those in the sham group (4.03 ± 1.20).

Subjective ratings of arousal of the pictures (selected from the no-reappraisal and reappraisal sessions) were examined using the two-way mixed-design ANOVA (regulation type × TMS group), and no significant interaction or main effect was found (Fig. 3*B*).

### fMRI results

#### ROI analysis

**Figure 4.**
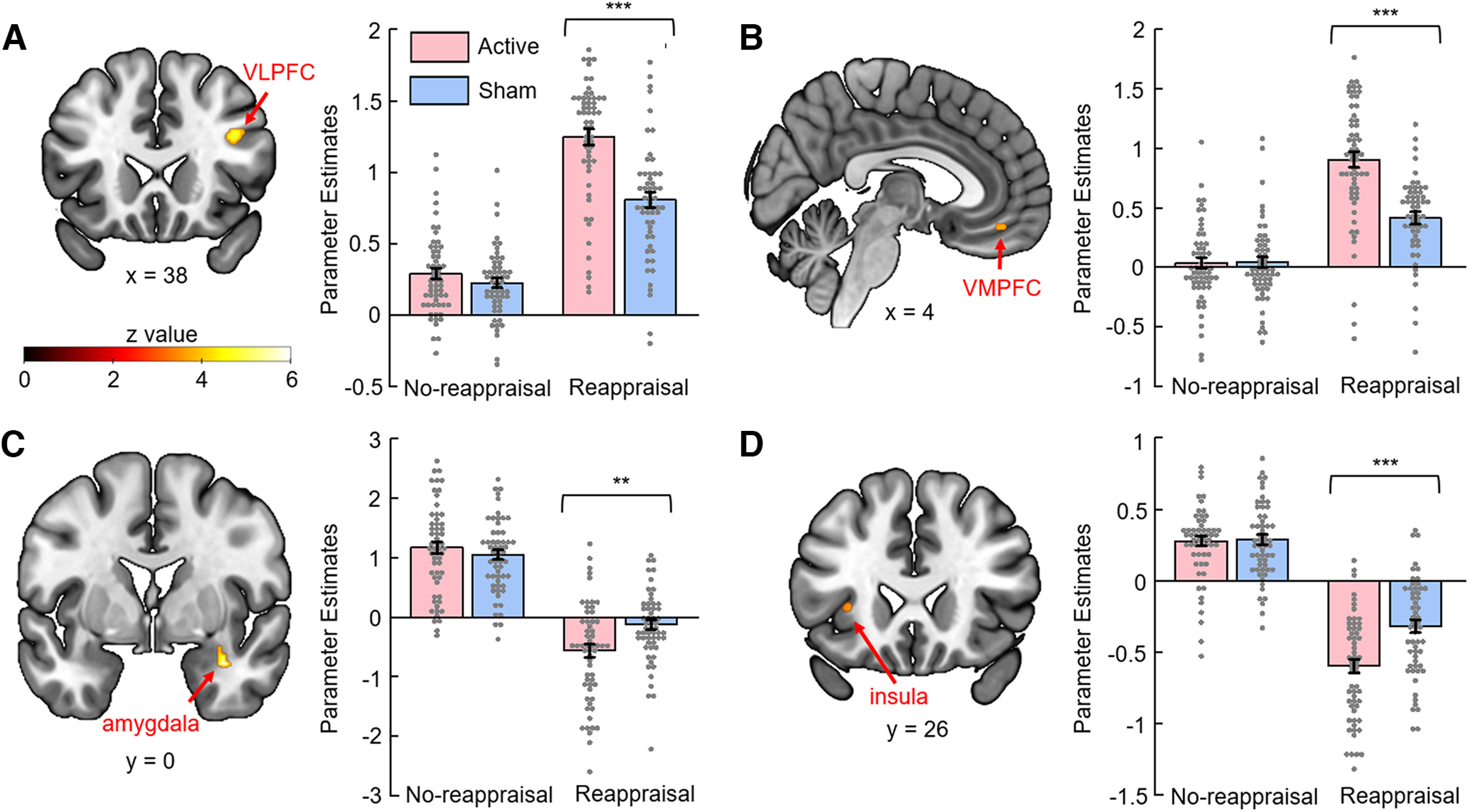
Brain activity (analyses of *a priori* ROI) showing a significant interaction effect between group (sham vs active TMS groups) and task condition (reappraisal vs no-reappraisal sessions). For brain activity maps, voxel-wise analyses were conducted within each ROI, and the surviving voxels (after performing FDR correction across all the voxels within the ROI) are displayed on the SPM canonical template. For bar charts, β weights were extracted from the averaged activity within each ROI. ***A***, Right VLPFC. ***B***, Right VMPFC. ***C***, Right amygdala. ***D***, Left insula. Error bars indicate SEM. **p < 0.01. ***p < 0.001.

The full factorial analysis demonstrated significant interaction effects between TMS group × regulation type in the right VLPFC, right VMPFC, right amygdala, and left insula (Fig. 4; Table 2). According to the ANOVA of mean parameter estimates for each ROI, these interactions were all driven by a significant group difference during the reappraisal condition, but not during the no-reappraisal condition. The clusters shown in the brain maps of Figure 4 are ROI voxel-wise level results. Bar plots in Figure 4 and the following ANOVA results reported in the text are ROI-level results.

**Table 2.** Clusters showing significant TMS group × regulation type interactions

Region	Cluster size (voxels)	*z* score	*p*	*x*	*y*	*z*
R VLPFC	62	4.97	0.005	38	18	26
R VMPFC	15	4.11	0.008	4	40	−16
R amygdala	25	4.85	<0.001	34	0	−24
L insula	15	3.80	0.010	−30	26	6

Significance was set at *p* < 0.012 (Bonferroni correction).

**Table 3.** Clusters showing the significant main effect of regulation type

Region	Cluster size (voxels)	*z* score	*p*	*x*	*y*	*z*
Contrast: reappraisal > no−reappraisal						
R VLPFC	262	5.18	<0.001	38	18	26
R VMPFC	51	5.01	<0.001	4	40	−16
Contrast: no−reappraisal > reappraisal						
R amygdala	81	5.85	<0.001	30	0	−24
L amygdala	6	4.85	0.021	−30	−2	−20
L insula	50	4.71	0.005	−30	24	6
R insula	3	3.31	0.098	32	26	2

The peak voxels were treated as the group maxima VOIs included in DCM model space. Significance was set at *p* < 0.012 (Bonferroni correction).

In particular, the analysis of the VLPFC ROI revealed a significant interaction between TMS group × regulation type (*F*_(1,115)_ = 19.90, *p* < 0.001, $\eta_{p}^{2}$ = 0.148). This was explained by the fact that, while the active TMS group (1.24 ± 0.42) showed enhanced activity compared with the sham TMS group (0.81 ± 0.40) in the reappraisal block (*F*_(1,115)_ = 31.48, *p* < 0.001, $\eta_{p}^{2}$ = 0.215), no significant group difference (active TMS group = 0.28 ± 0.28; sham TMS group = 0.22 ± 0.26) was observed in the no-reappraisal block (*F*_(1,115)_ = 1.59, *p* = 0.209, $\eta_{p}^{2}$ = 0.014).

The analysis of the VMPFC ROI revealed a significant interaction between TMS group × regulation type (*F*_(1,115)_ = 27.61, *p* < 0.001, $\eta_{p}^{2}$ = 0.194). This was explained by the fact that, while active TMS group (0.92 ± 0.51) showed enhanced activity compared with the sham TMS group (0.40 ± 0.40) in the reappraisal block (*F*_(1,115)_ = 36.75, *p* < 0.001, $\eta_{p}^{2}$ = 0.242), no significant group difference (active = 0.02 ± 0.36; sham = 0.04 ± 0.33) was observed in the no-reappraisal block (*F*_(1,115)_ = 0.14, *p* = 0.71, $\eta_{p}^{2}$ = 0.001).

The analysis of the amygdala ROI revealed a significant interaction between TMS group × regulation type (*F*_(1,115)_ = 9.47, *p* = 0.003, $\eta_{p}^{2}$ = 0.076). This was explained by the fact that, while active TMS group (−0.58 ± 0.85) showed reduced activity compared with the sham TMS group (−0.13 ± 0.60) in the reappraisal block (*F*_(1,115)_ = 11.31, *p* = 0.001, $\eta_{p}^{2}$ = 0.090), no significant group difference (active = 1.14 ± 0.76; sham = 1.03 ± 0.62) was observed in the no-reappraisal block (*F*_(1,115)_ = 0.69, *p* = 0.408, $\eta_{p}^{2}$ = 0.006).

The analysis of the insula ROI revealed a significant interaction between TMS group × regulation type (*F*_(1,115)_ = 12.57, *p* < 0.001, $\eta_{p}^{2}$ = 0.099). This was explained by the fact that, while active TMS group (−0.60 ± 0.36) showed reduced activity compared with the sham TMS group (−0.32 ± 0.33) in the reappraisal block (*F*_(1,115)_ = 19.11, *p* < 0.001, $\eta_{p}^{2}$ = 0.142), no significant group difference (active = 0.28 ± 0.26; sham = 0.29 ± 0.26) was observed in the no-reappraisal block (*F*_(1,115)_ = 0.05, *p* = 0.831, $\eta_{p}^{2}$ = 0.001).

For the main effect of regulation type, the no-reappraisal > reappraisal contrast identified group maxima in the right amygdala and left insula. The reappraisal > no-reappraisal contrast identified group maxima in the right VLPFC and right VMPFC. The location of these group maxima is reported in Table 3.

#### Whole-brain analysis

**Figure 5.**
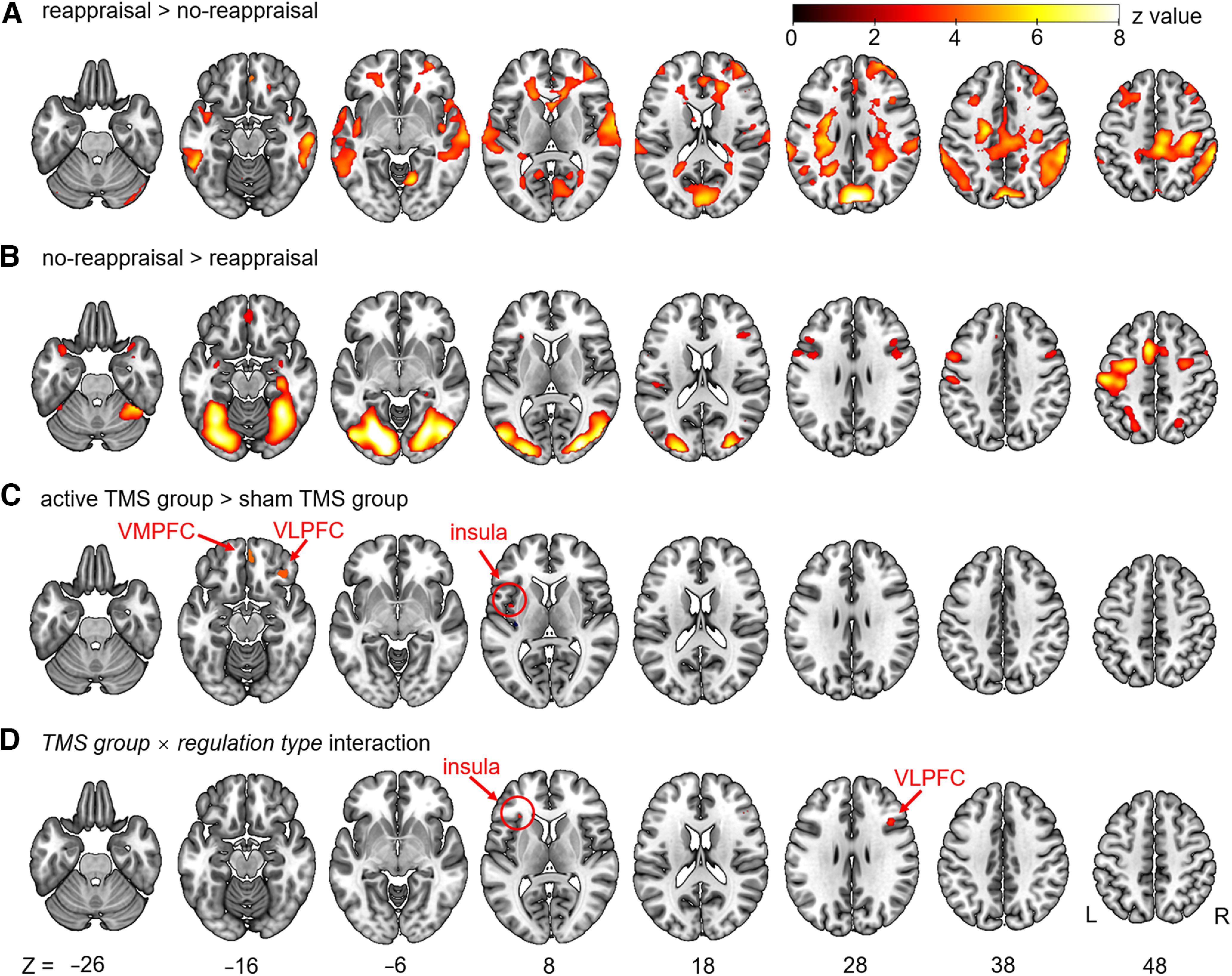
Results of the whole-brain analysis in all participants (*n* = 117). ***A***, Regions activated during reappraisal > no-reappraisal. ***B***, Regions activated during no-reappraisal > reappraisal. ***C***, Regions activated for the main effect of TMS: active TMS > sham TMS. ***D***, Regions showing a significant interaction effect between group (active TMS > sham TMS) and task condition (reappraisal > no-reappraisal). Data are thresholded at *p* < 0.05 (FDR-corrected with a minimum cluster size of 10 voxels, displayed on the SPM canonical template).

The main effect of the regulation type was observed in extensive brain regions in the frontal, temporal, parietal, and occipital lobes as well as the limbic system. Noticeably, the no-reappraisal > reappraisal contrast revealed significant brain clusters in the right amygdala and left insula, whereas the reappraisal > no-reappraisal contrast revealed significant brain clusters in the right VMPFC. The main effect of the TMS group revealed significant brain clusters in the right VLPFC, right VMPFC, and left insula. The peak activation in the VLPFC (*x* = 38, *y* = 30, *z* = −14; as identified in the contrast: active TMS > sham TMS) was 0.4 cm from the VLPFC site for TMS (*x* = 38, *y* = 34, *z* = −14). The interaction between TMS group × regulation type revealed significant brain clusters in the right VLPFC and left insula. The full results of whole-brain analyses are presented in Figure 5 and Table 4 (FDR-corrected).

**Table 4.** Results of the whole-brain analysis in all participants (*n* = 117)

Region	Cluster size (voxels)	*z* score	*p*	*x*	*y*	*z*
Contrast: reappraisal > no−reappraisal						
L cuneus	889	7.74	<0.001	0	−80	32
L middle cingulate cortex	470	7.03	<0.001	−18	−10	36
L caudate		5.62	<0.001	−24	−22	28
L crus 2 of cerebellar hemisphere	468	6.98	<0.001	−44	−68	−40
L crus 2 of cerebellar hemisphere		6.30	<0.001	−46	−56	−42
L crus 1 of cerebellar hemisphere		5.07	<0.001	−48	−62	−32
R postcentral gyrus	2103	6.70	<0.001	24	−44	66
R inferior parietal: supramarginal and angular gyri		6.17	<0.001	58	−38	48
R supramarginal gyrus		6.12	<0.001	60	−32	40
R precuneus	307	6.28	<0.001	2	−54	70
R paracentral lobule		6.28	<0.001	0	−42	74
R Heschl's gyri	521	5.89	<0.001	64	−2	6
R middle temporal gyrus		5.56	<0.001	60	−22	−16
R superior temporal gyrus		5.44	<0.001	66	−18	−4
R middle cingulate and paracingulate gyri	285	5.85	<0.001	4	−30	48
R supplementary motor area		5.68	<0.001	10	−22	50
R middle cingulate and paracingulate gyri		5.08	<0.001	2	−24	36
R lingual gyrus	68	5.84	<0.001	14	−62	−4
L inferior temporal gyrus	122	5.50	<0.001	−52	−42	−18
L middle temporal gyrus		5.03	<0.001	−50	−42	−10
R middle frontal gyrus	66	5.42	<0.001	28	56	28
R middle frontal gyrus		5.22	<0.001	38	52	22
L caudate	54	5.39	<0.001	−16	24	12
L supramarginal gyrus	70	5.35	<0.001	−66	−30	28
L supramarginal gyrus		4.96	<0.001	−62	−42	36
R middle frontal gyrus	31	5.21	<0.001	40	36	42
R middle frontal gyrus		4.78	<0.001	34	30	42
R VLPFC*^a^*	71	5.17	<0.001	38	18	26
R VLPFC*^a^*		4.21	<0.001	40	24	22
L cuneus	17	5.01	<0.001	−22	−52	28
R inferior temporal gyrus	16	5.00	<0.001	56	−42	−16
R caudate	25	5.00	<0.001	12	26	4
R middle frontal gyrus	14	5.00	<0.001	42	50	10
R precentral gyrus	19	4.94	<0.001	24	−14	66
R caudate	14	4.93	<0.001	4	16	8
R VMPFC*^a^*	27	4.86	<0.001	4	40	−16
Contrast: no−reappraisal > reappraisal						
L fusiform	5254	>8	<0.001	−24	−76	−8
L fusiform		>8	<0.001	−32	−58	−12
L middle occipital gyrus		>8	<0.001	−44	−74	2
R middle temporal gyrus	5156	>8	<0.001	48	−72	−2
R fusiform		>8	<0.001	30	−70	−10
R middle occipital gyrus		7.69	<0.001	30	−86	10
L supplementary motor area	3529	>8	<0.001	−6	2	54
L precentral gyrus		6.41	<0.001	−34	−16	56
L postcentral gyrus		6.37	<0.001	−50	−18	48
R amygdala*^a^*	114	4.99	<0.001	34	0	−24
R hippocampus		3.67	<0.001	26	−6	−22
Vermis_9	70	4.30	<0.001	−2	−56	−34
Vermis_9		3.13	0.001	6	−58	−38
L temporal pole: superior	104	4.09	<0.001	−36	8	−28
L amygdala*^a^*		3.46	<0.001	−32	−4	−16
L amygdala*^a^*		3.24	0.001	−30	2	−24
R precentral gyrus	252	4.09	<0.001	30	−4	48
R middle frontal gyrus 2		2.93	0.002	42	4	58
L precentral gyrus	331	4.08	<0.001	−52	4	38
L precentral gyrus		3.71	<0.001	−40	6	30
L precentral gyrus		3.13	0.001	−56	8	26
L insula*^a^*	126	3.87	<0.001	−30	22	2
L insula*^a^*		2.59	0.005	−36	16	6
L rolandic operculum	66	3.88	<0.001	−46	−24	20
R hippocampus	11	3.78	<0.001	26	−34	−4
L inferior parietal: supramarginal and angular gyri	306	3.72	<0.001	−20	−66	52
L inferior parietal: supramarginal and angular gyri		3.41	<0.001	−28	−48	46
R superior parietal gyrus	109	3.71	<0.001	22	−64	52
R insula*^a^*	67	3.62	<0.001	30	28	0
R precentral gyrus	346	3.43	<0.001	44	6	30
R precentral gyrus		3.09	0.001	50	8	40
L VLPFC	84	3.35	<0.001	−44	22	26
R temporal pole: superior temporal gyrus	30	3.24	0.001	36	16	−26
R rectus	50	3.19	0.001	2	44	−18
L insula*^a^*	11	3.00	0.001	−34	−20	14
Main effect of TMS active TMS > sham TMS						
R VLPFC*^a^*	78	4.22	<0.001	38	30	−14
R VMPFC*^a^*	32	3.88	<0.001	4	46	−15
L insula*^a^*	15	3.79	0.001	−34	−18	6
L insula*^a^*	34	3.71	0.003	−38	−2	6
L insula*^a^*				−38	−6	−2
Interaction effect TMS group × regulation type						
R VLPFC*^a^*	17	3.51	0.020	38	18	26
L insula*^a^*	11	3.44	0.021	−30	26	6

Data are thresholded at *p* < 0.05 (FDR-corrected with a minimum cluster size of 10 voxels), with MNI coordinates listed.

*^a^*Regions denoting task-specific responses matching hypothesis−driven ROIs.

#### DCM results

**Figure 6.**
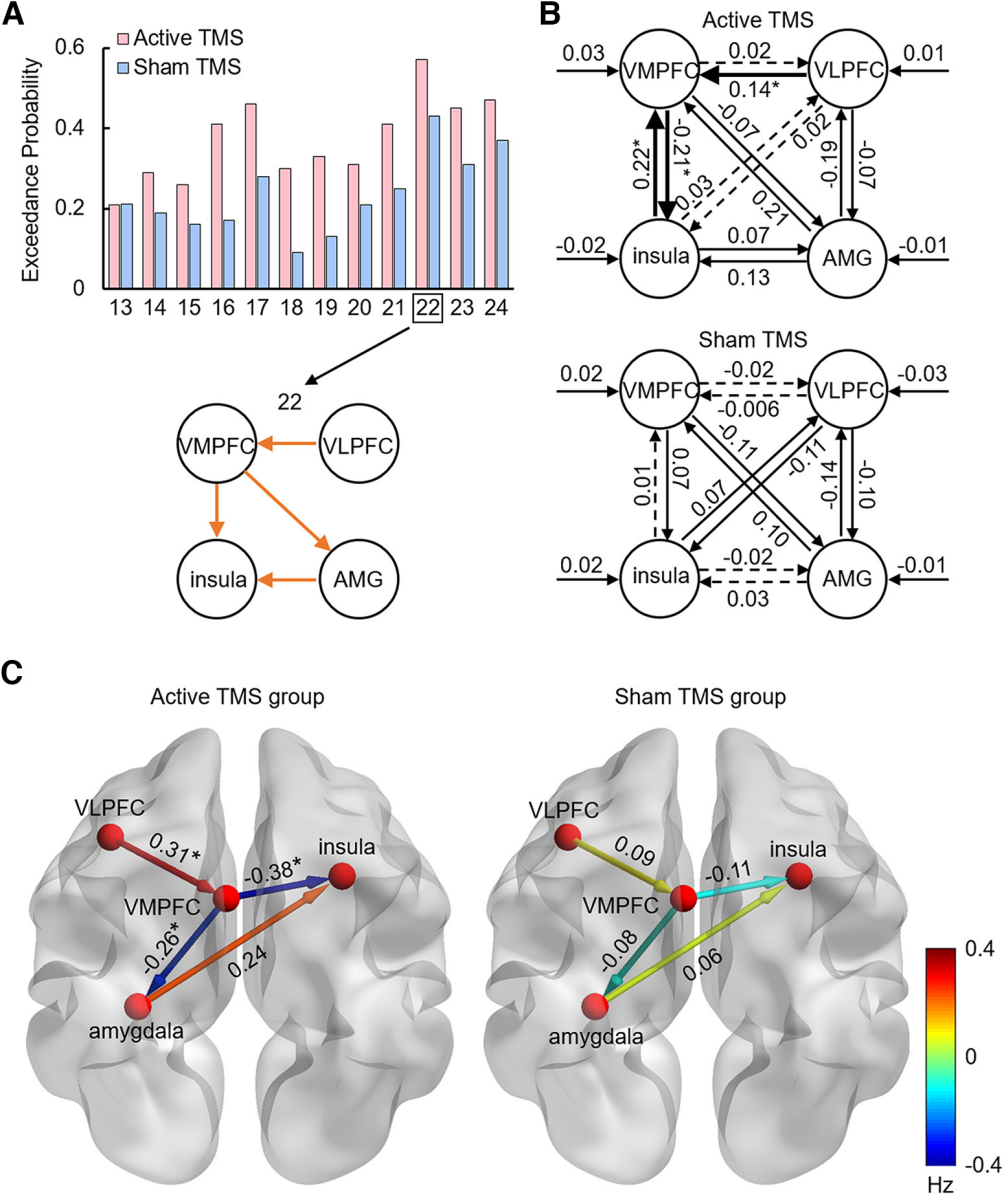
Results of Bayesian model selection and Bayesian model averaging. ***A***, Result of random-effects Bayesian model selection. Exceedance probability for all models in the indirect model family was calculated for the active and the sham TMS group, respectively. Model 22 provided the best fit for our data with an exceedance probability of 0.571 (active) and 0.432 (sham). In this model, connectivity from the VLPFC to the VMPFC, from the VMPFC to the amygdala (AMG) and insula, and from the amygdala to the insula are modulated by the emotion regulation effect (orange arrows). ***B***, The group-mean intrinsic parameters and driving inputs for active and sham TMS groups. Solid arrows indicate significant connectivity (posterior probability > 0.95). Dotted arrows indicate insignificant connectivity. Bolded arrows indicate increased excitatory or inhibitory connectivity in the active as compared with the sham TMS group (posterior probability > 0.95). ***C***, The group mean modulatory parameters of the two TMS groups. Nodes and connections are plotted on an adult brain model (BrainNet Viewer) (Xia et al., 2013). Color of the lines represents the magnitude of each connection. *Stronger connectivity in the active than the sham TMS group (posterior probability > 0.95).

Direct and indirect models were first compared at the family level using random-effects Bayesian model selection. As a result, the indirect model family, which had VLPFC-to-VMPFC-to-subcortical connections modulated by the ER effect, was a better explanation of the data with a total exceedance probability of 0.995 (active) and 0.992 (sham), as compared with the direct model family with a total exceedance probability of 0.006 (active) and 0.013 (sham). Then, the 12 DCM models within the indirect model family were compared using random-effects Bayesian model selection; model 22 outperformed the other models with an exceedance probability of 0.571 (active) and 0.432 (sham; Fig. 6*A*).

Finally, Bayesian model averaging analysis was performed on model 22 (the winning model). Parameter estimates for intrinsic connections and driving inputs are shown in Figure 6*B*. Group statistic of DCM parameters shows that there are significant connection differences between the active and sham TMS groups. For the intrinsic connections, the active TMS group has increased excitatory connectivity from the VLPFC to the VMPFC, from the insula to the VMPFC, but increased inhibitory connectivity from the VMPFC to the insula, as compared with the sham TMS group (Fig. 6*B*). The modulatory effect of ER (reappraisal vs no-reappraisal) significantly differed on three predefined connections between the two groups: the active TMS group had stronger excitatory connectivity from VLPFC to VMPFC and stronger inhibitory connectivity from VMPFC to the insula and amygdala, as compared with the sham TMS group (Fig. 6*C*). All DCM parameters of the two TMS groups are shown in Table 5.

**Table 5.** Parameters of the model with the best fit (model 22), including intrinsic connections, modulation of intrinsic connections, and driving inputs

Item	Active TMS group (*N* = 59)			Sham TMS group (*N* = 58)			*t*	*p*(FDR)
	Mean ± SE (Hz)	*t*	*p*(FDR)	Mean ± SE (Hz)	*t*	*p*(FDR)		
Intrinsic connectivity								
VLPFC to VMPFC	0.14 ± 0.03	2.35	<0.001	<−0.01 ± 0.01	0.97	0.152	4.57	<0.001
VLPFC to amygdala	−0.07 ± 0.03	−1.07	<0.001	−0.10 ± 0.01	−1.76	<0.001	2.91	0.084
VLPFC to insula	0.02 ± 0.01	0.02	0.489	−0.11 ± 0.02	−1.21	<0.001	0.63	0.192
VMPFC to VLPFC	0.02 ± 0.01	0.16	0.154	−0.02 ± 0.01	−0.06	0.119	0.52	0.133
VMPFC to amygdala	−0.07 ± 0.01	−2.13	<0.001	−0.11 ± 0.01	−2.21	<0.001	0.12	0.657
VMPFC to insula	−0.21 ± 0.04	−2.11	<0.001	0.07 ± 0.02	0.98	0.001	−3.22	<0.001
Amygdala to VLPFC	−0.19 ± 0.05	−2.08	<0.001	−0.14 ± 0.02	−1.91	<0.001	−0.20	0.591
Amygdala to VMPFC	0.21 ± 0.04	3.13	<0.001	0.10 ± 0.01	2.87	<0.001	2.10	0.098
Amygdala to insula	0.13 ± 0.03	1.73	<0.001	0.03 ± 0.02	0.97	0.136	3.02	0.077
Insula to VLPFC	0.03 ± 0.01	1.02	0.080	0.07 ± 0.02	1.99	<0.001	−1.05	0.210
Insula to VMPFC	0.22 ± 0.06	5.03	<0.001	0.01 ± 0.01	1.01	0.312	9.77	<0.001
Insula to amygdala	0.07 ± 0.01	1.76	<0.001	−0.02 ± 0.01	−0.68	<0.001	0.19	0.057
Modulation by emotion regulation								
VLPFC to VMPFC	0.31 ± 0.07	6.33	<0.001	0.09 ± 0.03	2.18	<0.001	8.99	<0.001
VMPFC to amygdala	−0.26 ± 0.09	−7.60	<0.001	−0.08 ± 0.02	−1.88	<0.001	−9.54	<0.001
VMPFC to insula	−0.38 ± 0.05	−9.37	<0.001	−0.11 ± 0.03	−2.69	<0.001	−7.89	<0.001
Amygdala to insula	0.24 ± 0.02	4.16	<0.001	0.06 ± 0.01	3.15	0.079	6.81	0.007
Driving input								
to VLPFC	0.01 ± 0.01	2.17	<0.001	−0.03 ± 0.01	−2.19	<0.001	1.90	0.095
to VMPFC	0.03 ± 0.01	2.29	<0.001	0.02 ± 0.01	1.89	<0.001	0.010	0.977
to amygdala	−0.01 ± 0.01	−1.56	<0.001	−0.01 ± 0.01	−1.47	<0.001	−0.082	0.855
to insula	−0.02 ± 0.01	−2.05	<0.001	0.02 ± 0.02	2.00	<0.001	−3.01	0.075

Independent-samples *t* test was performed (two-tailed) between active and sham TMS groups. One-sample *t* test was performed within active or sham stimulation groups. All *p* values were corrected for multiple comparisons using the FDR method.

#### Brain-behavioral correlations

**Figure 7.**
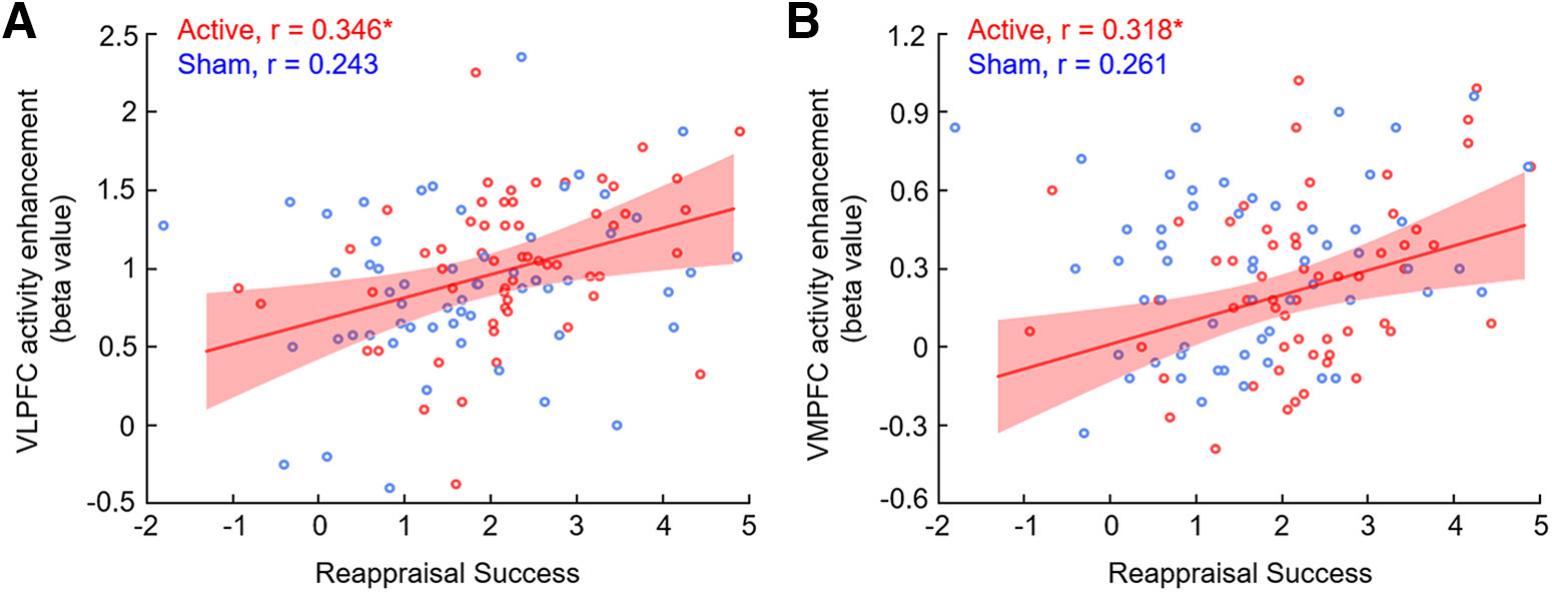
Correlation between reappraisal success and brain activity changes because of emotion regulation (reappraisal vs no-reappraisal). Higher level of reappraisal success is associated with higher enhancement of the activity in the VLPFC (subplot ***A***) and in the VMPFC (subplot ***B***) in the active TMS group. *p < 0.05, set FDR correction for multiple comparisons. Shaded areas represent 95% confidence band of the regression line.

Two correlations were significant (Table 6), showing that higher regulation success correlated positively with activity enhancement from the no-reappraisal to the reappraisal condition in the VLPFC (Fig. 7*A*; *r* = 0.346, *p*(FDR) = 0.028) and VMPFC (Fig. 7*B*; *r* = 0.318, *p*(FDR) = 0.028) in the active TMS group. Those correlations were significant or marginally significant in the sham TMS group but did not survive the FDR correction (VLPFC: *r* = 0.243, *p*(FDR) = 0. 188; VMPFC: *r* = 0.261, *p*(FDR) = 0.132). Fisher *r*-to-*z* transformation (Zar, 1996) revealed no significant difference for the correlation coefficients between the two TMS groups in the VLPFC (*z* = 0.59, *p* = 0.278) and VMPFC (*z* = 0.33, *p* = 0.371).

**Table 6.** Correlations between participants' reappraisal success and brain activity during reappraisal versus no-reappraisal

ROIs	Sham group (*N* = 58)			Active group (*N* = 59)		
	*r*	*p*	*p*(FDR)*^a^*	*r*	*p*	*p*(FDR)*^a^*
R VLPFC	0.243	0.066	0.188	0.346	0.007	0.028*
R VMPFC	0.261	0.047	0.132	0.318	0.014	0.028*
R amygdala	−0.011	0.641	0.561	−0.115	0.463	0.105
L insula	−0.102	0.421	0.641	−0.201	0.079	0.463

*^a^*Corrected using the FDR correction method.

*p < 0.05.

## Discussion

Using combined TMS-fMRI setups, the present study aimed to answer two questions regarding voluntary ER. Results confirmed our hypothesis that the TMS-induced VLPFC facilitation led to enhanced activity in the prefrontal network and attenuated activity in the subcortical regions, and that the activated VLPFC intensified the prefrontal-subcortical couplings via the VMPFC.

*Is voluntary ER dependent on the VLPFC, and is downregulating negative emotion implemented by the VLPFC's suppression of the subcortical affective system?* To answer this question, we observed in the brain activity data that the TMS-evoked neural stimulation not only emerged at the TMS target (i.e., VLPFC), but also propagated to connected remote regions, including the VMPFC, amygdala, and insula. Specifically, TMS caused enhanced activity in the VLPFC and VMPFC under the reappraisal, but not in the no-reappraisal condition. While the VLPFC is considered a key node in the prefrontal cognitive control system (Urry et al., 2006; Johnstone et al., 2007; Delgado et al., 2008; Ochsner et al., 2012), the VMPFC is regarded essential in both cognitive control and emotional value integration during ER (S. H. Kim and Hamann, 2007; Winecoff et al., 2013). Both these frontal regions significantly activated during the downregulation of negative emotions, consistent with previous meta-analysis findings (Diekhof et al., 2011; Kohn et al., 2014; but see Buhle et al., 2014). In support of the prefrontal finding, brain-behavioral correlation in the active TMS group revealed that ER-modulated enhancement of activity in the VLPFC and VMPFC was positively correlated with reappraisal success.

In addition to prefrontal areas, we also observed that TMS caused reduced activity in the amygdala and insula under the reappraisal versus the no-reappraisal condition. The amygdala and insula are core regions in the subcortical affective system responsible for emotion generation and representation (Ochsner et al., 2012). They both belong to the salience network (Seeley et al., 2007), responsible for detecting and orienting attention toward threatening (typically the amygdala) (Paton et al., 2006) and painful (typically the insula) (Eisenberger, 2012; W. Li et al., 2021) stimuli. The observed attenuated activity in the two regions is in line with previous subcortical findings during reappraisal of negative affective stimuli (Goldin et al., 2008; Ochsner and Gross, 2008; Frank et al., 2014). Beyond existing knowledge acquired using neuromodulation or neuroimaging in isolation, this study provides causal perturb-and-measure evidence regarding the voluntary ER being critically dependent on the VLPFC, and that downregulating negative emotion is a process that involves the prefrontal control system suppressing the subcortical affective system. Using fMRI to examine the TMS effect, we uncovered that (1) voluntary ER is influenced by the VLPFC and its interactions with other brain regions, specifically the VMPFC and subcortical affective areas; and (2) the opposite neural changes in prefrontal (enhanced) and subcortical (attenuated) regions are not a byproduct of voluntary ER; instead, this prefrontal-subcortical activity intrinsically and causally contributes to the ER effect.

*How does the prefrontal top-down control system connect with the subcortical affect system?* To answer this question, we investigated brain connectivity and found increased activity in three ER-modulated pathways (i.e., excitatory VLPFC-to-VMPFC, inhibitory VMPFC-to-amygdala, and inhibitory VMPFC-to-insula pathways) because of VLPFC facilitation. First, it is not surprising that the VMPFC is the downstream region affected by the VLPFC activation. Both regions are anatomically connected (Ongur and Price, 2000), and their functional coupling is predictable for ER success (Cha et al., 2016; Morawetz et al., 2016). Second, the inhibitory effect of the VMPFC on the amygdala has been well identified during ER in many studies (for reviews, see Myers-Schulz and Koenigs, 2012; Andrewes and Jenkins, 2019); for example, stronger inhibitory coupling between them predicts more adaptive stress responses during reappraisal (Urry et al., 2006; Johnstone et al., 2007). Third, enhanced VMPFC activity has been associated with lessened insula activity and reduced pain experience (Eisenberger et al., 2011); this VMPFC-insula connectivity is predictable for the ability to downregulate stressful/fearful responses (Hart et al., 2018; Dutcher et al., 2020). More importantly, the DCM results revealed a crucial engagement of the VMPFC within the VLPFC-subcortical network, thus supporting the indirect pathway model of the ER circuit (Hiser and Koenigs, 2018). Previous neuroimaging studies usually used threatening pictures from the International Affective Picture System (Lang et al., 2005) and revealed that the VMPFC is the “intermediate station” involved in reappraisal on the LPFC-to-amygdala pathway (e.g., Urry et al., 2006; Johnstone et al., 2007; Silvers et al., 2017; Steward et al., 2021). One of the contributions of this study is that we evoked social pain and identified that the inhibitory VMPFC-insula coupling is essential for the VLPFC's modulation of the insula during reappraising (the insula is more sensitive to painful than fearful stimuli). It might seem surprising that the meta-analysis by Buhle et al. (2014) did not identify the VMPFC in the ER circuit. In our opinion, this could be because the VMPFC is engaged during both voluntary and automatic ER; thus, its activation might not be apparent when contrasting reappraisal to nonreappraisal (viewing naturally) conditions (Buhle et al., 2014). This study used TMS to amplify the neural changes within the voluntary ER circuit; therefore, the “bridge” role of the VMPFC is highlighted under the reappraisal versus nonreappraisal contrast. Furthermore, this “perturb-and-measure” approach overcomes the correlational nature of fMRI data (Bergmann et al., 2021), helping us to identify brain regions that causally support reappraisal (i.e., the VLPFC and VMPFC) and those that are modulated by reappraisal (i.e., the amygdala and insula). A recent TMS study highlighted a direct white matter pathway between the VLPFC and the amygdala using diffusion MRI (Sydnor et al., 2022). However, considering the imperfect correspondence between structural and functional connectivity (Suárez et al., 2020), we argue that the Sydnor et al. (2022) study and the current DCM findings are not necessarily contradictory because, while we reveal the indirect pathway in the context of emotion reappraisal, Sydnor et al. (2022) have identified a structural direct pathway in general.

The laterality of brain activity observed in our study warrants discussion. Our ROI analysis identified brain activity showing interaction and main effects of ER primarily in the right hemisphere, including the right VLPFC, VMPFC, and amygdala. However, we also observed increased activity in the left insula, which appears contradictory to the right lateralization pattern. The right lateralization of most brain activities could be attributed to the use of right hemisphere TMS, but the left-lateralized insula finding is intriguing. In previous studies, the left insula shows greater activation than the right insula during perception of others' emotions (Wicker et al., 2003; Singer et al., 2004; Caria et al., 2010) and social-emotional judgments (Quarto et al., 2016). Also, a review found left insula dominance in emotion perception (Duerden et al., 2013), suggesting a lateralization of affective processing. The task used in this study may have specifically engaged left insula processes associated with emotion perception.

The above uncovered ER circuit is not only important for understanding the neural systems underlying reappraisal, but it is also valuable for translational research. First, the finding that the VLPFC-VMPFC coupling works as a top-down regulation pathway during voluntary ER provides novel avenues for clinical practice. For diagnosis, both the structure and function of LPFC (for review, see Zilverstand et al., 2017) and VMPFC, as well as their connectivity, are potential specific brain characteristics for identifying and assessing ER impairments. For treatment, neuromodulation and neurofeedback methods aiming to facilitate both regions or intensify their positive coupling are potential protocols to enhance voluntary ER ability in patients with mood and anxiety disorders. Second, our behavioral results demonstrate that the TMS-facilitated ER persisted for at least half an hour, as revealed by more positive rating of pictures 30 min following the ER implementation in the active compared with the sham TMS group. This finding corroborates the evidence found in our prior work showing that ER ability enhancement produced by single TMS session could prolong for at least 0.5-1 h (He et al., 2020b; Zhao et al., 2021). Meanwhile, Lapate et al. (2017) have found that the TMS-modulated VLPFC produced changed emotional stimuli evaluation, which could last for 3 d. In line with these findings, future translational studies may benefit from examining this prolonged ER effect in clinical populations using multiple-session TMS protocols to transfer the TMS-induced neuroplasticity into long-term ER benefits.

In conclusion, this study revealed that the VLPFC serves as an essential brain region to support the voluntary ER process, while the downstream propagation of reappraisal unfolds as a “chain reaction” from the prefrontal control network into the emotion integrative area VMPFC and to the subcortical affective regions. Although the uncovered neural circuit of voluntary ER is somewhat primitive (comprising only four cortical and subcortical regions), we believe this is a critical first step in building a sophisticated neural model of ER, including the DLPFC, dorsomedial PFC, supplementary motor area, periaqueductal gray, and parietal and temporal regions using combined brain investigation techniques.
